# A Cell-Regulatory Mechanism Involving Feedback between Contraction and Tissue Formation Guides Wound Healing Progression

**DOI:** 10.1371/journal.pone.0092774

**Published:** 2014-03-28

**Authors:** Clara Valero, Etelvina Javierre, José Manuel García-Aznar, María José Gómez-Benito

**Affiliations:** 1 Multiscale in Mechanical and Biological Engineering (M2BE), Aragón Institute of Engineering Research (I3A), University of Zaragoza, Zaragoza, Spain; 2 Centro Universitario de la Defensa de Zaragoza, Academia General Militar, Zaragoza, Spain; University of California, San Diego, United States of America

## Abstract

Wound healing is a process driven by cells. The ability of cells to sense mechanical stimuli from the extracellular matrix that surrounds them is used to regulate the forces that cells exert on the tissue. Stresses exerted by cells play a central role in wound contraction and have been broadly modelled. Traditionally, these stresses are assumed to be dependent on variables such as the extracellular matrix and cell or collagen densities. However, we postulate that cells are able to regulate the healing process through a mechanosensing mechanism regulated by the contraction that they exert. We propose that cells adjust the contraction level to determine the tissue functions regulating all main activities, such as proliferation, differentiation and matrix production. Hence, a closed-regulatory feedback loop is proposed between contraction and tissue formation. The model consists of a system of partial differential equations that simulates the evolution of fibroblasts, myofibroblasts, collagen and a generic growth factor, as well as the deformation of the extracellular matrix. This model is able to predict the wound healing outcome without requiring the addition of phenomenological laws to describe the time-dependent contraction evolution. We have reproduced two in vivo experiments to evaluate the predictive capacity of the model, and we conclude that there is feedback between the level of cell contraction and the tissue regenerated in the wound.

## Introduction

Wound healing is an intricate process that combines biological, chemical and mechanical signals for collective cell function. Normal wound healing evolves over three overlapping phases: inflammation, proliferation and remodeling [1], [2]. When homeostasis is reached a few hours after wounding, the inflammatory phase begins with neutrophil and macrophage cell invasion and debridement of the wound site [1]. Subsequently, these cell types secrete and/or recruit specialized biochemical growth factors, such as TGF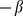, PDGF and MDGF which control the subsequent stages of the healing process. Re-epithelialization of the wound also occurs during the inflammation phase. Epithelial cells proliferate and move to the top of the wound. During the proliferative phase, biochemical mediators recruited during the inflammatory phase control the migration, proliferation and bio-signal production of fibroblasts and endothelial cells. Fibroblasts degrade the initial fibrin blood clot [3] and secrete collagen type III, creating a new extracellular matrix at the wound site that is more resistant than the blood clot but has inferior mechanical properties than the undamaged tissue. The inferior mechanical properties of the granulation tissue are due to, among other factors, the random alignment of the new secreted collagen fibers. Matrix remodeling occurs over a period of months, increasing the proportion of collagen type I and causing the formation of scar tissue that resembles healthy skin. Endothelial cells follow migrating fibroblasts and re-establish the vascular system that provides the oxygen and nutrients required for cell function. There is evidence that both biochemical factors (such as TGF-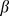) [4] and mechanical stimuli induce the differentiation of fibroblasts into myofibroblasts [5], leading to wound contraction.

Tissue cells are anchored to a substrate and use their acto-myosin system to exert and transmit contractile forces to their surroundings [6]. Mechanical stimuli are known to influence several cellular processes such as migration, differentiation and orientation [7]–[12]. Moreover, there is evidence that the mechanical stimulus that regulates these processes is the stiffness of the substrate that surrounds the cells [5], [8], [13]–[17]. To clarify this phenomenon, Mitrossilis et al. [15] demonstrated that cells on elastic substrates modify their activity according to the substrate stiffness. Their in-vitro experiments demonstrated that the forces exerted by cells increase as the substrate becomes stiffer [15], and that a saturation force level is reached. Cells are anchored to the substrate through focal adhesions and show different behaviors depending on the mechanical properties of the substrate; they are stronger on stiffer surfaces.

Computational modeling makes it possible to reproduce and evaluate the wound healing progress under different conditions. To provide valuable predictions, the healing process needs to be fully understood and translated into mathematical equations. Moreover, computational models can be of great aid for the discussion of certain biological hypotheses. Early wound healing models [18]–[20] could predict the evolution of epidermal wounds. Murray et al. [19] developed the first biochemical model of wound contraction in one dimension, which was used to study the evolution of a cellular species and the extracellular matrix (ECM) density and displacement. Sherratt et al. [20] proposed a biochemical model in which cell proliferation and migration are dictated by a generic growth factor. These models have been further developed to incorporate biophysical evidence acquired from in vitro or animal models. Olsen et al. [21], [22] proposed the first mechano-chemical model of wound contraction, in which the major events in fibroplasia and wound contraction are taken into account, including the addition of a new cellular species under study, the myofibroblasts, which have a relevant role in wound contraction. A thorough analysis of these model equations enabled the establishment of the effect of chemical net production on the occurrence of fibroproliferative disorders, particularly the effect of a permanently contracted state. Adam [18] investigated the occurrence of non-healing wounds and the so-called critical size defect with a simple model that describes the evolution of a generic growth factor activating cell proliferation at the wound edge. Olsen's model [21] has been recently revised by different authors [23]–[25]. These works incorporate the decreased mechanical properties of the granulation tissue and combine for the first time the coupled actions of chemical and mechanical factors on the fibroblast to induce myofibroblast differentiation, although the studies differ in the mechanical stimulus used to drive the differentiation. Both works suggest that differentiation is guided by stress. Whereas Javierre et al. [23] claim that the stress that guides the process is the force exerted by the cells, Murphy et al. [24] propose that this stress comes from the elastic component of the ECM. Additionally, Javierre et al. [23] investigated the effect of wound shape on the contraction kinetics, whereas Murphy et al. [24], [25] focused on a more detailed representation of the biochemical signaling of wound contraction. Furthermore, Javierre et al. [23] considered a unique growth factor that regulates differentiation and collagen production, whereas Murphy et al. [25] included the chemical kinetics of two different growth factors (PDGF and TGF-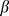) separately.

Several cellular mechanisms have been found to be driven by the stiffness of the substrate that surrounds the cells and not by the stresses that the cells support [8], [15]. Thus, we propose a differentiation mechanism that combines both chemical factors and a mechanical stimulus, as performed in previous works, but we assume that the mechanical stimulus that regulates the differentiation process is the ECM deformation, which depends directly on the ECM stiffness.

Therefore, in this work, we propose a unified constitutive theory consistent with experimental observations of individual and collective cell populations. This theory is based on a rigidity sensing mechanism that cells use to control the level of contraction that they exert on the ECM to drive its deformation. This deformation of cells is able to indirectly regulate the progression of different cellular events, such as cell differentiation and tissue formation.

## Results

### Cell traction forces are modulated in response to the rigidity of the surrounding ECM

Early works on wound contraction assumed that cells exert a constant traction force (denoted by 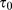 or 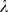) on the ECM. This constant traction force is subsequently scaled (or modulated) by the ECM density (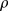) and the cellular densities of fibroblasts (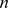) and myofibroblasts (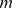). Traditionally, a linear relationship between cell-induced stresses and cell densities is assumed. Moreover, the myofibroblasts-enhanced traction forces are modeled through the proportionality factor 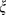. Finally, the most significant difference between these models arises in the term for the ECM density. This term represents the different properties on the involved tissues (the wound and the partly recovered and healthy skin) during contraction progression. All of these hypotheses have been included through different phenomenological laws, such as [21], [23]

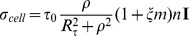
(1)


or [25]

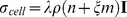
(2)


These expressions aim to induce an increase in the cell-exerted stresses in the middle of the wound, creating a stress gradient between the wound and the surrounding healthy tissue. Note that this gradient is therefore dependent on the initial conditions of the model with respect to the ECM density and the cell populations.

Other authors instead proposed phenomenological laws that are non-linear to the cell population. In those cases, the stresses exerted by cells tend to become saturated due to contact inhibition and competition for ECM binding sites at high cell densities [26]

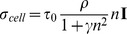
(3)


However, there are multiple experiments that suggest that the cellular capacity to exert traction forces on the ECM strongly depends on the ECM stiffness [27]. Therefore, in this work we propose a purely mechanical and self-regulated traction force dependent on the ECM stiffness through
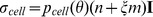
(4)


In this expression, we consider the role of the ECM stiffness through 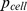, which denotes the force that a cell exerts depending on the volumetric strain (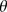) of the ECM [28]. Unlike other models, we do not explicitly include the ECM density in the expression for 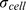. However, the ECM density does play an indirect role in cell-induced stresses through the value of 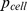 (see File S1). As the collagen density increases, the tissue becomes stiffer [29], regulating the volumetric strain of the tissue 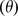, which in turn defines the value of 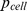 and the stresses exerted by the cells on the ECM. We consider also that 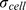 depends on the concentration of fibroblasts and myofibroblasts, with a term 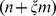 similar to the one proposed by Murphy et al. [25]. Contractile forces exerted by fibroblasts can initiate wound closure and myofibroblasts are known to contribute to the transmission of these contraction forces [29], [30]. Furthermore, any of these species can be present in the absence of the other. Hence, when one of them is not present, the other one can still generate stress [31]. This basic effect is not included in most of the previous models, in which myofibroblasts are not considered [26] or in which the generated forces are always zero in the absence of fibroblasts [21], [23].

**Figure 1:**
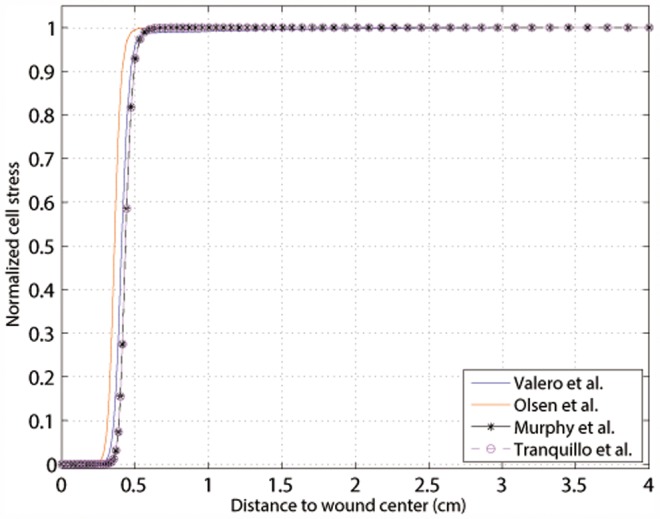
Normalized cell stress (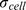) distributions created by different laws [21], [25], [26] and the proposed model at the beginning of wound contraction. The wound has a radius of 0.5 cm. Every law produces a similar stress distribution despite dependence on different variables.

Figure 1 shows that the initial stress distributions (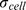) exerted by the cells in the wounded and unwounded tissues are very similar in all considered theories. Therefore, we can conclude that all previous phenomenological laws represent similar behavior and thus, we can find a clear biophysical interpretation of this behavior which is the mechanosensing mechanism provided by cells.

### Fibroblast to myofibroblast differentiation is driven by cell deformation

We assume that fibroblasts differentiate into myofibroblasts in response to the strain (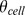) supported by the fibroblasts. This strain is the same as the strain of the ECM because we consider cells and the ECM to occupy the same domain and because they both support the same strain as the compatibility condition.

This assumption for fibroblast differentiation into myofibroblasts is based on the following phenomena. When a wound occurs and healing events are activated, fibroblasts exert contractile forces as a mechanosensing mechanism. Thus, fibroblasts shrink the external domain of the wound, and consequently, the inner part of the wound is extended. This effect forces the fibroblasts inside the wound to stretch. To overcome this effect, we hypothesize that fibroblasts differentiate into myofibroblasts regulated by the passive stretching that fibroblasts support inside the wound due to the fibroblast contraction in the external part of the wound. This result is consistent with experimental evidence that establishes that mechanical forces such as stretching can drive fibroblasts to differentiate toward a myofibroblast phenotype [29], [32], [33]. When the population of myofibroblasts inside the wound also exerts contractile stresses, the full contraction of the wound occurs, and the differentiation of fibroblasts to myofibroblasts is stopped.

**Figure 2:**
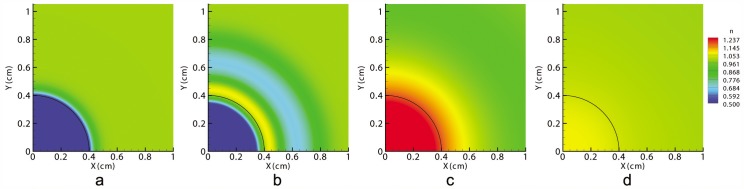
Fibroblast distributions in the tissue at t = 0 (a), at the beginning of the contraction (b), at halfway through the healing time (c) and at healing time (d). The black line denotes the edge between the initial wound and the surrounding skin.

**Figure 3:**
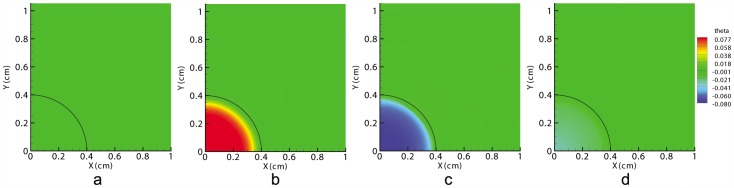
Volumetric deformations in the tissue at t = 0 (a), at the beginning of the contraction (b), at halfway through the healing time (c) and at healing time (d). The black line denotes the edge between the initial wound and the surrounding skin.

**Figure 4:**
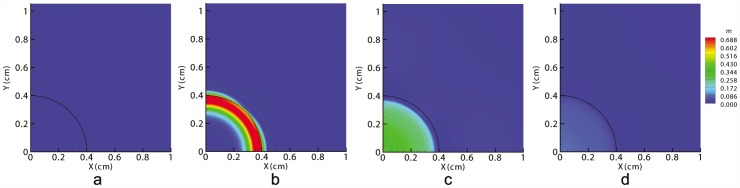
Myofibroblasts distributions in the tissue at t = 0 (a), at the beginning of the contraction (b), at halfway through the healing time (c) and at healing time (d). The black line denotes the edge between the initial wound and the surrounding skin.

Therefore, the first observable consequence of an injury is the distraction of the wound due to fibroblasts distribution (Figure 2). This deformation causes the ECM volumetric strain, 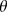, to be positive at the wound center and negative (although very close to zero) in the surrounding undamaged tissue (Figure 3), which in turn causes myofibroblasts to appear inside the wound, close to its edge (Figure 4).

As time passes, fibroblasts and myofibroblasts accumulate inside the wound, creating the necessary traction forces to overcome the passive stretch of the wound. From that moment on, the wound contracts, and the sign of ECM volumetric deformation gradually changes from positive to negative from the wound boundary inward (see Figure 3).

Therefore, there are two different behaviors in the wound caused by the non-uniform cell and matrix densities. Cells can deform, contracting the matrix, or cells can be stretched due to the matrix deformation. Therefore, fibroblasts contract the ECM near the wound edge and stretch the wound center. This stretching is also included in the fibroblasts that are inside the wound site, and it regulates their differentiation into myofibroblasts (see Figure 4).

Thus, the proposed differentiation mechanism implies that there is no differentiation from fibroblasts to myofibroblasts in the healthy skin. This outcome is physically coherent, as myofibroblasts appear only inside the wound [31]. The use of volumetric tensile strains to differentiate provides a biophysical explanation for a phenomenon that has been previously simulated in a phenomenological way [21], [23], [25], [26].

### Comparative analysis of the predictive ability of the model with in-vivo experiments

The proposed mechano-chemical model makes it possible to study the evolution of the wound from two different perspectives. First, we analyze the deformation of the wound during its contraction. However, the contraction of the wound is accompanied by the synthesis and deposition of new tissue, which fills the wound space. Hence, we also analyze the healing of the wound in terms of collagen density. Collagen does not fill the wound completely until several months or years have passed [1]. Hence, we consider the wound to be healed when its collagen density is at least 75% of the density in healthy skin. It is safe to assume that when this threshold of collagen concentration is reached, the skin has mostly recovered its mechanical properties and functionality.

**Figure 6:**
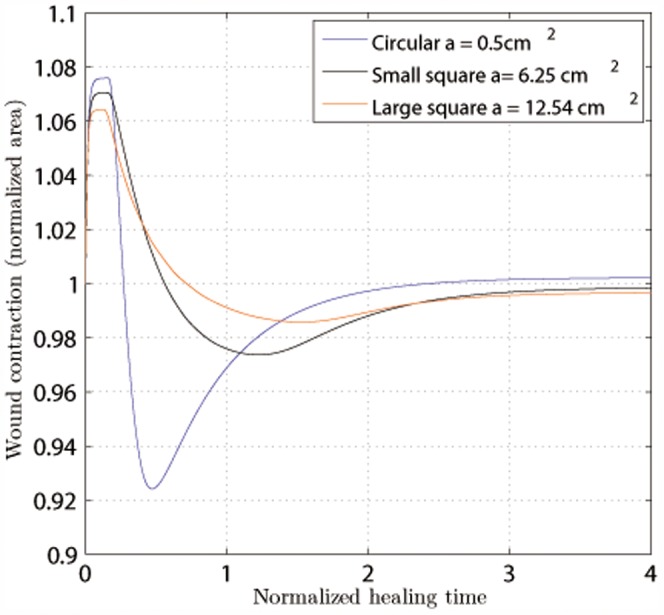
Wound contraction as a function of time for the three studied geometries.

We have reproduced the wound geometries used by Roy et al. [34] and McGrath and Simon [35] in animal models. Roy et al. [34] considered a circular wound of area of 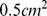 in pigs, whereas McGrath and Simon [35] considered square wounds with areas of 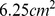 and 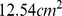 in rats. The area of the tissue initially occupied by the wound is used to determine the contraction pattern of the considered geometries. The temporal evolution of this area (normalized with respect to its initial size) is presented in Figure 6. The release of the skin stresses is a direct consequence of the injury, which causes a fast increase in the wound area. However, as time passes, the wound contracts due to the forces exerted by the cells, finally a size similar to the initial size. Based on the considered geometries, we can conclude that a larger wound size leads to a smoother transition between the distraction-contraction regimes.

**Figure 5:**
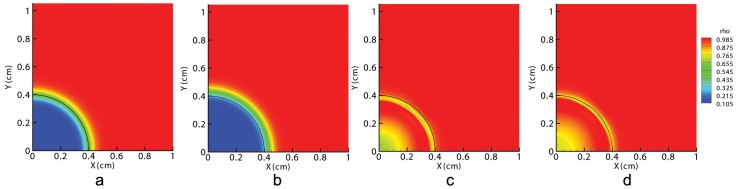
Collagen distributions in the tissue at t = 0 (a), at the beginning of the contraction (b), at halfway through the healing time (c) and at healing time (d). The black line denotes the edge between the initial wound and the surrounding skin.

**Figure 7:**
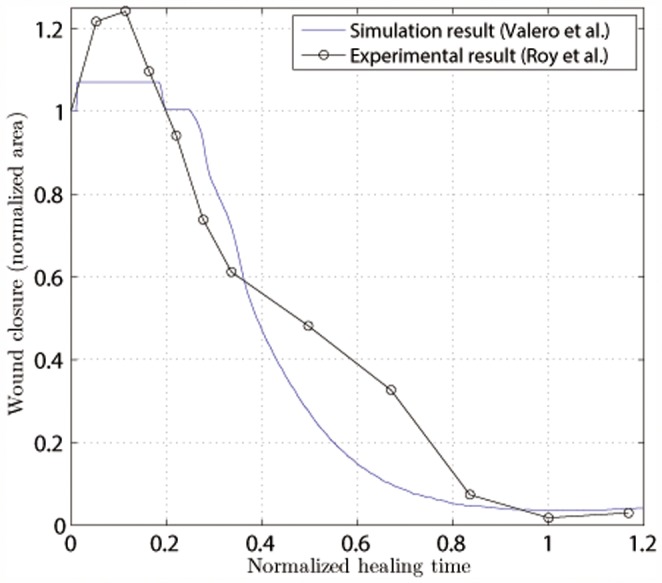
Normalized wound area as a function of the normalized healing time for a circular wound with a radius of 0.4 Comparison with the experimental work of Roy et al.[34].

**Figure 8:**
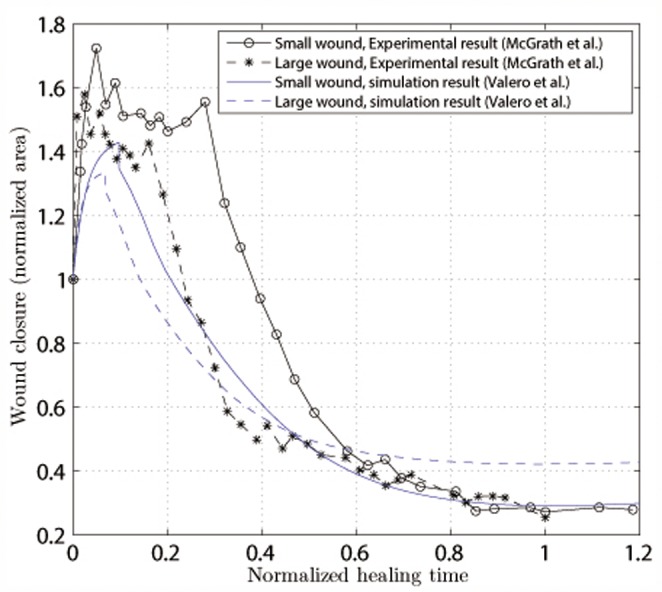
Normalized wound area as a function of the normalized healing time (time 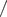
healing time) for two square wounds with areas of 6.25 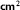 and 12.54 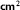. Solid lines refer to the small wound, and dashed lines refer to the large wound. Comparison with the experimental work of McGrath and Simon [35].

The healing pattern for the considered geometries is obtained via the temporal evolution of the wound area (normalized to its initial size). As introduced before, we consider the wound to be all parts of the tissue with less than 75% of the collagen density of the undamaged tissue. This variable is presented in Figures 7 and 8, for the experimental results [34], [35]. In both cases, we accurately capture the healing kinetics at the early stages of the healing process. Collagen appears rapidly at the early stages of healing and it stabilizes after reaching its maximum value of wound closure (Figure 5).

Roy et al. [34]observed the evolution of an ischemic wound and a non-ischemic wound in a pig. We have used the non-ischemic wound for comparison with our results as we simulate a wound in non-pathological skin. We present wound closure as a function of time. We simulate wound healing in humans, and the experiments were performed on different animal species. When comparing the results, it should be take into consideration that each species has different time parameters due to their different cellular and tissue kinetics [36]. Hence, for each set of experiments (simulations and in vivo) and in order to adjust the differences in time scales, we fix the healing time as the moment when the maximum healing is reached. For the small circular geometry (area of 0.5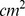) analyzed by Roy et al. [34] (Figure 7) the numerical simulation closely predicts the closure rate at the latter stages of the healing process. We see that the initial distraction stage lasts for approximately 10% of the healing time and that the healing curves in both cases follow a similar pattern, reaching a similar healing level. In both cases, almost complete healing is obtained.

For the larger geometries, we simulate the experiments of McGrath and Simon [35] (Figure 8), in which square wounds of different areas were considered (sizes of 6.25 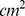 and 12.54 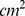). We observe that the numerical simulation underestimates the percentage of wound closure (for the time period considered). As in the experimental work, we found that the larger wounds heal slightly less than smaller wounds. We also found that the elastic modulus of the rat's skin is one order of magnitude smaller than the elastic modulus of the pig's skin.

The results show differences between the two cases based on several reasons. First, the mechanical properties of the two animal species have different orders of magnitude. Moreover, the wound sizes should be considered to be of different orders of magnitude in the two experimental works. Although the wound studied by [34] can be considered small relative to the animal size, the wound studied by [35] has a large size compared with the animal size. This fact greatly influences the healing process. In [35] the wound probably affects the muscular zone with movement, which impairs greater healing.

When studying square wounds, we find that the healing pattern tends to soften the curvature of the wound. This phenomenon has been previously observed in other biological processes, such as bone ingrowth in bone scaffolds. This phenomenon corroborates the idea that wound healing is a mechanically driven process [37].

## Discussion

The economic and social impact of the treatment of chronic wounds calls for an integrated and multidisciplinary approach to the problem. Mathematical modeling and computer simulation should be used as additional tools to gain a better understanding of the intricate biochemical and mechanical processes behind wound healing.

In this work, we present a mechano-chemical wound healing model with two main novelties that distinguish it from previous models. We postulate that the main phenomena that occur during wound healing involves cells and are regulated by mechanical stimulation. Thus, we propose to update the phenomenological laws with physical evidence-based laws for fibroblast differentiation and the cell-exerted stresses.

This work provides a mechanical theory of wound contraction that is consistent with the cell function experimental observations [15], [16] and with wound healing in animal models [34], [35]. The proposed model generalizes from previous models [19], [21]–[23], [25] with a cell-regulatory mechanism that handles ECM rigidization and its impact on cell function. Our model provides similar results to those of previous works, but we have proposed a formulation of cell traction generation and fibroblasts differentiation based on a biophysical hypothesis instead of a phenomenological assumption. Taking these modifications into account, the model can help to clarify our knowledge of regenerative phenomena.

The effect of additional phenomena (naturally produced by the organism or externally induced) could be analyzed with the current model definition, either changing the mechanical properties of the affected tissues to the ones measured for each pathology or varying specific model parameters. This is the case of certain pharmacological therapies [38] or genetic mutations involving modifications in the tissue properties, mostly rigidization, that could be studied using the present model. Moreover, it will be possible the study of certain pathologies such as pressure ulcers [39] or fibroproliferative disorders [40], which have a high mechanical component and modify the natural evolution of wound healing. In pressure ulcers, oxygen flow is impaired due to an excessive pressure in the tissue that comprises blood vessels [39]. Moreover, the hydrostatic pressure becomes negative in the skin area subjected to pressure leading to negative volumetric strains. Thus, fibroblasts differentiation into myofibroblasts will be inhibited once the ulcer has begun, and traction forces generated by fibroblasts will not be enough for closing the wound. The opposite cases are fibroproliferative disorders such as keloids and hypertrophic scars, which appear due to an excessive collagen production during healing [40]. It is also known that the appearance of these disorders is promoted by mechanical forces [41], [42]. An overexpression of collagen will cause an excessive tension in the tissue that surrounds the wound, which will also produce an excessive fibroblast differentiation. Moreover, it is known that the collagen type created in every process is different, having different stiffness properties, which could be included in the model.

Several assumptions and simplifications were needed to formulate and implement this model. First, although wound healing is a three-dimensional process, we considered a two-dimensional simplification in our work. We considered a plane stress approach, neglecting wound depth and assuming that the deformation on the plane is constant.

Most of the existing models [19], [21], [25] make great simplifications about wound geometry, considering only one-dimensional axisymmetric wounds. This simplification is useful for analyzing theoretical wounds but cannot be applied to simulate real and more complicated wounds. Thus, we follow [23] and consider a two-dimensional model that could be extended to three dimensions, which would be more appropriate to reproduce the real behavior of wounds in the skin.

Other simplifications in the model are made when defining cell stresses. Although we have assumed that stresses are mainly due to the cell activity, other sources such as patient motion could generate stress. We have also considered the volumetric cell strain as the mechanical variable that regulates cell biology, however, other mechanical variable such as the deviatoric cell strain or the principal cell strain could have been considered. Moreover, the influences of other factors such as chemical growth factors are indirectly included in the model through the cellular kinetics.

Regarding the numerical results, the wound does not reach complete healing during the studied time. In fact, complete healing is never achieved after a wound occurs [1]; the tissue keeps recovering for months or years. Moreover, a critical size defect (CSD) is known to exist [18]. This CSD is different for each animal species and denotes the wound size above which a wound will not heal during the animal's lifetime.

Although computational simulations reproduce an ideal situation, there are several external and unpredictable factors in animal experiments that should be considered. Moreover, the mechanical properties of the skin vary depending on the location on the body. The skin can displace and contract in different ways depending on how it is oriented relative to tension lines.

Scarring is the step that follows wound contraction, and the model presented here will be of great aid for preliminary qualitative prediction of the scarring level. Moreover, the model makes it possible to study different factors that regulate scarring, such as wound size and shape, the animal species and the mechanical properties of the skin.

## Materials and Methods

In this work, we model the temporal evolution of different cellular species (fibroblasts and myofibroblasts), chemicals (a generic growth factor with the combined effects of PDGF and TGF-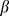 on (myo)fibroblasts and the collagen density) and extracellular matrix deformation [30], [43]. The cellular and chemical species densities are obtained from a conservation law
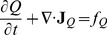
(5)


where 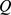 denotes the cellular/chemical species, 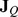 denotes its net flux over the domain of interest (which may include terms representing random dispersal -migration or diffusion-, directed migration (chemotaxis), and may also include a passive convection term due to ECM deformation), and 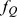 denotes net production. The matrix deformation is obtained from the conservation of linear momentum
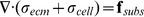
(6)


where 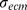 denotes the passively resistant ECM stress, 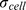 denotes the ECM stress due to the cells-ECM adhesions and 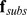 denotes the ECM-substrate anchoring forces that resist ECM deformation.

This work follows the model proposed by Javierre et al. [23], based on the well-established model of Olsen et al. [21]. We consider the presence of two cellular species, fibroblasts (n) and myofibroblasts (m), embedded in a collagen (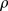) matrix and guided by the presence of a chemical growth factor (c). We also consider the matrix displacements (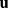) as a primary variable in the model (see File S1).

Fibroblasts, connective tissue cells found in the skin, are the main cellular species involved in wound contraction. The main functions of fibroblasts are the synthesis of connective tissue in response to injury and remodeling of the collagen ECM by the exertion of traction forces [44]. Fibroblasts are motile cells that migrate by random dispersal, chemotaxis and passive convection caused by the ECM displacements. Hence, their net flux term can be written as
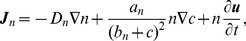
(7)


where 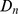 denotes the fibroblast diffusion rate, and 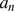 and 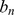 are chemotaxis-related parameters. The parameter values can be found in Table 1 and Table 2.

**Table 1:** List of model parameters related to fibroblasts and myofibroblasts kinetics.

Parameter	Description	Value	Observations
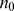	fibroblasts density in undamaged dermis	10^4^ cells/cm 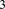	[21]
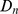	fibroblasts diffusion rate	2 10^−2 ^cm 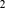 /day	[27] 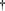
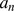	together with 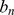 determines the maximal chemotaxis rate per unit of GF concentration	4 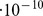 g/cm day	[23]
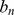	GF concentration that produces 25% of the maximal chemotactic response	2 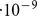 g/cm 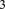	[23]
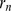	fibroblasts proliferation rate	0.832day 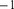	[27]
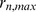	maximal rate of GF induced fibroblasts proliferation	0.3 day 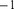	[23]
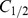	half-maximal GF enhancement of fibroblasts proliferation	10 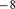 g/cm 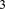	[21]
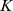	fibroblasts maximal capacity in dermis	10 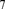 cells/cm 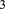	[21]
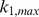	maximal rate of fibroblasts differentiation	0.8 day 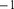	[23]
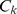	half-maximal GF enhancement of fibroblasts differentiation	10 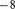 g/cm 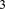	[23]
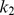	myofibroblasts desdifferentiation rate	0.693 day 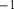	[23]
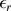	proportionality factor	0.5	[21]

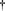
 Adjusted to fit reported migration rate with a traveling wave model.

**Table 2:** List of model parameters related to collagen and growth factor kinetics.

Parameter	Description	Value	Observations
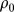	collagen concentration in undamaged dermis	0.1 g/cm 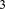	[21]
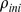	initial collagen concentration in the wound	10 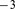 g/cm 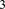	[21]
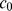	GF concentration in the wound	10 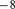 g/cm 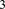	[21]
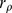	collagen production rate	7.59 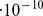 g 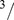 cm 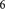 cell day	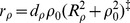
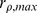	maximal rate of GF induced collagen production	7.59 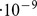 g 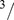 cm 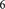 cell day	[21]
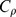	half-maximal GF enhancement of collagen synthesis	10 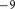 g/cm 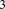	[21]
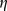	proportionality factor	2	[21]
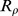	half-maximal collagen enhancement of ECM deposition	0.3 g/cm 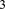	[21]
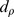	collagen degradation rate per unit of cell density	7.59 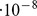 cm 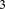 /cell  day	[21]
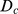	GF diffusion rate	5 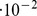 cm 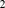 /day	[21]
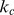	GF production rate per unit of cell density	7.5 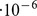 cm 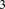 /cell day	[23] 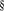
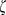	proportionality factor	1	[21]
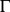	half-maximal enhancement of net GF production	10 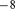 g/cm 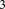	[21]
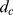	GF decay rate	0.693 day 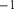	[23]

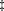
 Determined collagen degradation kinetics to remain in equilibrium away from the wound.

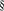
 Downestimated to prevent fibro-proliferative disorders [22] with the used GF decay rates.

Fibroblasts kinetics is determined by their proliferation, differentiation into myofibroblasts, differentiation back from myofibroblasts and apoptosis. The novelty with respect to Javierre et al.[23] is the signal that triggers fibroblast differentiation. The ability of fibroblasts to sense the strain in the ECM [45] and the regulation of their differentiation to myofibroblasts by mechanical loads [30], [31], [46] are well known. Hence, we consider whether the differentiation process is driven by the deformation of the tissue where the cells are allocated instead of depending on the mechanical stress of the matrix itself [29]. We also maintain the hypothesis that this differentiation is also enhanced by different growth factors (e.g.,PDGF and TGF-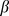) [31], [47]. Hence, fibroblast differentiation into myofibroblasts can be expressed through the term
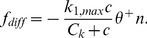
(8)


where 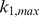 denotes the maximal rate of fibroblast differentiation and 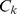 regulates the influence of the growth factor during differentiation.

Fibroblasts differentiate into myofibroblasts under the influence of TGF-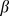 and the resulting phenotype is able to exert and maintain higher contractile forces in the tissue [46]. Fibroblast differentiation into myofibroblasts occurs when the ECM has a positive volumetric strain, which is denoted by 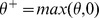. In this situation, cells are able to exert forces on the tissue, which means that the strain is not mainly caused by the tissue itself. Myofibroblasts are smooth muscle-like cells [31], which means that they are not motile and that their flux is only due to passive convection. Myofibroblast evolution is mainly due to proliferation, differentiation from fibroblasts, inverse differentiation to fibroblasts and apoptosis [48].

Cells in the skin are embedded in the ECM, with the main components being collagen fibers produced by fibroblasts. Hence, we model the ECM density through the collagen density. Collagen fibers are non-motile, and hence their net flux term is expressed in terms of the passive convection of the skin.

Following the model of Olsen et al. [21] we consider the role of fibroblasts and myofibroblasts in collagen synthesis [31], [49]. Furthermore, collagen production is enhanced by the presence of growth factors such as TGF-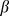 [50].

The wound-healing process is regulated by several growth factors. Collagen-matrix contraction is regulated by PDGF [30] among other factors, and fibroblast differentiation is driven by TGF-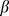 [31]. In this work, we consider a unique growth factor that regulates these processes for simplicity. The net flux of the growth factor is due to passive convection and also to diffusion through the tissue. Growth factor production is regulated by fibroblasts and myofibroblasts, following [23].

After a wound occurs, there is an instantaneous elastic response of the skin that causes the wound edge to retract, increasing the wound size. During this distraction process, the pre-stress of the skin is relaxed. Hence, the factors determining the change in wound geometry are purely mechanical. The time scale at which stress liberation occurs (on the order of minutes) is much smaller than the time scale at which cellular events such as migration, differentiation, proliferation and matrix production occur (on the order of days). Therefore, we assume that cells do not have time to influence the process, except by the death of cells due to the wounding process.

Once wound distraction has reached equilibrium, we consider the resulting wound geometry. The deformations accumulated until full wound distraction are also felt by the cells and they activate the mechanosensing mechanism controlling wound contraction. The skin is assumed to be a viscoelastic material [23], [26].

The second major novelty of this work with respect to earlier works [21], [23], [25], [26] rests on the expression for cell-induced stresses. The ECM deformation is obtained from the conservation of linear momentum (Equation (6)), where 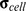 denotes the cell-exerted stresses. If we denote the traction force exerted by one fibroblast as 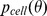 [28], we can write the cell-induced stresses as
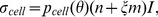
(9)


where the parameter values are included in Table 3.

**Table 3:** List of model parameters related to the mechanical behavior of cells and ECM.

Parameter	Description	Value	Observations
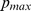	maximal cellular active stress per unit of ECM	10 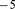 N  g 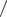 cm 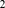 cell	[23]
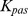	volumetric stiffness moduli of the passive components of the cell	2 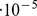 N  g 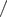 cm 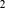 cell	[28]
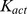	volumetric stiffness moduli of the actin filaments of the cell	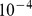 N  g 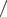 cm 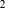 cell	[28]
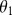	shortening strain of the contractile element	-0.6	[23]
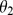	lengthening strain of the contractile element	0.5	[28]
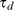	half-maximal mechanical enhancement of fibroblast differentiation	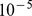 N  g 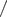 cm 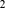 cell	[23]
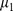	undamaged skin shear viscosity	200 N day/cm 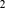	[23]
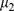	undamaged skin bulk viscosity	200 N day/cm 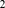	[23]
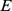	undamaged skin Young's modulus	3.34–33.4 N/cm 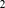	[51]
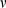	undamaged skin Poisson's ratio	0.3	[51]
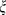	myofibroblasts enhancement of traction per unit of fibroblasts density	10- cm 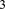 /g	[21]
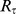	traction inhibition collagen density	5 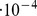 g/cm 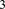	[21]
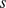	dermis tethering factor	10 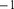 N/cm  g	Estimated

Finally, we consider the observation that the ECM-substrate anchoring forces resisting ECM deformation are proportional to the tissue displacement and to the ECM density.

## Supporting Information

File S1 **Description of the Mechano-chemical Model and the Model Implementation.**
(PDF)
