# Correction: A cell-regulatory mechanism involving feedback between contraction and tissue formation guides wound healing progression

**DOI:** 10.1371/journal.pone.0101714

**Published:** 2014-06-25

**Authors:** The PLOS ONE Staff

There are errors in the Funding section. The correct funding information is as follows: This research was supported by the Spanish Ministry of Economy and Competitiveness (project DPI2012-32880 and grant BES2010-037281). The financial support of the European Union through the European Regional Development Fund (project DPI2012-32880) and the European Research Council (project ERC-2012-StG 306751) is gratefully acknowledged. The funders had no role in study design, data collection and analysis, decision to publish, or preparation of the manuscript.

## References

[1] Valero C, Javierre E, García-Aznar JM, Gómez-Benito MJ (2014) A Cell-Regulatory Mechanism Involving Feedback between Contraction and Tissue Formation Guides Wound Healing Progression. PLoS ONE 9(3): e92774 doi:10.1371/journal.pone.0092774 24681636 10.1371/journal.pone.0092774 PMC3969377

