# Supplementary material for: A Cell-Regulatory Mechanism Involving Feedback between Contraction and Tissue Formation Guides Wound Healing Progression
Source: PLoS One. 2014 Mar 28;9(3):e92774. doi: 10.1371/journal.pone.0092774 (PMC3969377; doi:10.1371/journal.pone.0092774)
Supplement: File S1 — Description of the Mechano-chemical Model and the Model Implementation. (PDF) [file pone.0092774.s001.pdf]

Supplementary Material

This work follows the model proposed by [1], based on the well established model of [2]. We consider the presence of two cellular species, fibroblasts (n) and its phenotype myofibroblasts (m), embedded in a collagen (ρ) matrix and guided by the presence of a chemical growth factor (c). The density of these species and substances together with the ECM displacements ($\mathbf{u}$) are the model variables.

Mechano-chemical model

The cellular species (fibroblasts and myofibroblasts) and chemical substances (growth factor and collagen) densities are obtained from a conservation law

$$\frac{\partial Q}{\partial t} + \nabla \cdot \mathbf{J}_Q = f_Q \tag{1}$$

where Q denotes the cellular/chemical species, $\mathbf{J}_Q$ denotes its net flux over the domain of interest (that may include terms due to random dispersal -migration or diffusion-, directed migration -chemotaxis-, but contains a passive convection term due to the extracellular matrix (ECM) deformation), and f_Q its net production. The matrix deformation is obtained from the conservation of linear momentum

$$\nabla \cdot \left(\boldsymbol{\sigma}_{ecm} + \boldsymbol{\sigma}_{cell}\right) = \mathbf{f}_{subs} \tag{2}$$

where $\boldsymbol{\sigma}_{ecm}$ denotes the passive resistant ECM stress, $\boldsymbol{\sigma}_{cell}$ the ECM stress due to the cells-ECM adhesions and $\mathbf{f}_{subs}$ the ECM-substrate anchoring forces resisting ECM deformation.

Species concentrations

Fibroblasts are connective tissue cells found in skin and the main cellular species involved in wound contraction. The main functions of fibroblasts are the synthesis of connective tissue in response to injury and the remodeling of the collagen extracellular matrix (ECM) by exerting traction forces on it [3]. Fibroblasts are motile cells whose migration is due to random dispersal, chemotaxis and passive convection caused by the ECM displacements. Hence, its net flux term can be written as

$$\mathbf{J}_n = -D_n \nabla n + \frac{a_n}{(b_n + c)^2} n \nabla c + n \frac{\partial \mathbf{u}}{\partial t}, \tag{3}$$

where all the parameters are listed in Table 1.

Fibroblasts production is due to proliferation, differentiation into myofibroblasts and differentiation back from myofibroblasts. We propose a similar expression to the one presented by [1] but with a different differentiation term. It is well known that fibroblasts sense the strains in the ECM [4], and the differentiation to myofibroblasts is regulated by mechanical loads [5–7]. Hence, we consider that the differentiation process is driven by the deformation of the tissue where the cells are allocated instead of depending on the mechanical stress of the matrix [8]. Moreover, the differentiation process is also enhanced by different growth factors (PDGF,TGF-β) [7, 9]. Hence, the fibroblasts net production is given by the expression

$$f_n = \left(r_n + \frac{r_{n,max} c}{C_{1/2} + c}\right) n \left(1 - \frac{n}{K}\right) - \frac{k_{1,max} c}{C_k + c} \theta^+ n + k_2 m. \tag{4}$$

Fibroblasts differentiate into myofibroblasts under the influence of TGF-β and this resulting phenotype is capable of exerting and maintaining higher contraction forces in the tissue [6]. We propose a new differentiation mechanism guided by the cells volumetric strain. We consider that fibroblasts are able to differentiate into myofibroblasts only when their volumetric strain is negative, that is, when fibroblasts

are under compression. In this situation cells are able to exert forces in the tissue. Myofibroblasts are smooth-muscle like cells [7], which means that they are not motile and its flux is uniquely due to passive convection and can be written as,

$$\mathbf{J}_m = m\frac{\partial \mathbf{u}}{\partial t}. \tag{5}$$

Myofibroblasts evolution is mainly due to proliferation, differentiation from fibroblasts and inverse differentiation to fibroblasts and apoptosis [10]. Hence, myofibroblasts net production can be written as follows

$$f_m = \epsilon_r\Big(r_n + \frac{r_{n,max}c}{C_{1/2}+c}\Big)m\big(1-\frac{m}{K}\big) + \frac{k_{1,max}c}{C_k+c}\theta^+ n - k_2 m. \tag{6}$$

Cells in the skin are embedded in the ECM, whose main components are the collagen fibers, which are produced by fibroblasts and myofibroblasts. Hence, we model the ECM density through the collagen density. Collagen fibers are non motile, and hence their net flux is expressed in terms of the passive convection of the skin

$$\mathbf{J}_\rho = \rho\frac{\partial \mathbf{u}}{\partial t}. \tag{7}$$

Following [2] we consider the role of fibroblasts and myofibroblasts on collagen synthesis [7,11]. Furthermore, collagen production is enhanced by the presence of growth factors like TGF-β [12],

$$f_\rho = \Big(r_\rho + \frac{r_{\rho,max}c}{C_\rho+c}\Big)\frac{n+\eta_b m}{R_\rho^2+\rho^2} - d_\rho\big(n+\eta_d m\big)\rho. \tag{8}$$

The wound healing process is regulated by several growth factors. Collagen-matrix contraction is regulated by PDGF [5] among others and fibroblasts differentiation is driven by TGFβ [7]. In this work, we consider a unique generic growth factor that regulates these processes for simplicity. The net flux of growth factor is due to passive convection and diffusion through the tissue. This can be written as

$$\mathbf{J}_c = -D_c\nabla c + c\frac{\partial \mathbf{u}}{\partial t}. \tag{9}$$

The growth factor production is regulated by fibroblasts and myofibroblasts. Considering a simplification of the expression proposed by [2] and following [1] it can be written as

$$f_c = \frac{k_c(n+\zeta m)c}{\Gamma + c} - d_c c \tag{10}$$

where all the parameter values are included in Table 2.

Mechanosensing and mechanotransduction mechanism

This work proposes a new expression for calculating the stresses exerted by cells during wound healing. The new expression presented in the main manuscript is

$$\sigma_{cell} = p_{cell}(\theta)\,(n+\xi m)\,\mathbf{I} \tag{11}$$

where the traction force per cell, p_{cell}, is a function of the ECM volumetric deformation, θ. In this expression we consider the stiffening effect of the ECM through p_{cell}, that depends on the volumetric strain of the tissue [13]. Traction force per cell was introduced by Moreo et al. [13] and is defined as follows,

$$p_{cell}(\theta) \quad = \quad \frac{K_{act}p_{max}}{K_{act}\theta_1 - p_{max}}\Big(\theta_1 - \theta\Big)\chi_{[\theta_1,\theta^*]}(\theta) + \frac{K_{act}p_{max}}{K_{act}\theta_2 - p_{max}}\Big(\theta_2 - \theta\Big)\chi_{(\theta_1,\theta^*]}(\theta) + \; K_{pas}\theta \tag{12}$$

where all the parameters and their values are included in Table 3.

In this expression the two mechanisms that a cell uses to generate the stress are considered. The actin mechanism only actuates between some deformation limits, while the passive mechanism is always activated.

Initial distributions of cells and substances

Initial species distributions have been set according to experimental observations. The wound site is occupied by hematoma tissue with low mechanical stiffness and null cell population at the beginning of the analysis. However, we consider that the healthy tissue is saturated with fibroblasts, collagen and growth factor. Nevertheless, the initial density of myofibroblasts in the healthy tissue is zero, as they appear only where there has been damage.

Constitutive relation for the skin material

The skin was assumed as a viscoelastic material [1, 14]. For this kind of materials the passive resistant ECM stress $\boldsymbol{\sigma}_{ecm}$ is defined

$$\boldsymbol{\sigma}_{ecm} = \mu_1\frac{\partial\boldsymbol{\varepsilon}}{\partial t} + \mu_2\frac{\partial\theta}{\partial t}\mathbf{I} + \frac{E}{1+\nu}\Big(\boldsymbol{\varepsilon} + \frac{\nu}{1-2\nu}\theta\mathbf{I}\Big), \tag{13}$$

where the skin's Young's modulus (E) evolves following the expression $E = E_0\frac{\rho}{\rho_0}$, that considers a stiffer ECM as the collagen density increases. All the model parameters are listed in Table 3.

Model implementation

The problem has been solved using the finite element analysis and implemented using an Abaqus User Subroutine ®. Usual shape functions are used to interpolate the values of the primary unknowns (that is, n, m, ρ, c and $\mathbf{u}$) from their nodal values. Time derivatives are approximated using a generalized trapezoidal method.

Weak formulation

Equations (7) and (11) can be written equivalently using Gauss' theorem to obtain the weak formulation of the problem

$$\int_\Omega \frac{\partial Q}{\partial t}\upsilon_Q d\Omega - \int_\Omega \mathbf{J}_Q \cdot \nabla\upsilon_Q d\Omega = \int_\Omega f_Q\upsilon_Q d\Omega - \int_{\partial\Omega} \mathbf{J}_Q \cdot \mathbf{n}_\perp \upsilon_Q d\Gamma \tag{14}$$

$$\int_\Omega \boldsymbol{\sigma} : \frac{1}{2}\Big(\nabla\boldsymbol{\upsilon} + \nabla\boldsymbol{\upsilon}^T\Big)d\Omega = \int_\Omega \mathbf{f}_{subs}\cdot\boldsymbol{\upsilon}d\Omega \; + \int_{\partial\Omega}\mathbf{t}\cdot\boldsymbol{\upsilon}d\Gamma \tag{15}$$

where $\boldsymbol{\sigma}$ denotes the sum of $\boldsymbol{\sigma}_{ecm}$ and $\boldsymbol{\sigma}_{cell}$. υ_Q and $\boldsymbol{\upsilon}$ represent the weighting functions and $\mathbf{n}_\perp$ denotes the normal vector pointing outwards Ω and $\mathbf{t} := \boldsymbol{\sigma}\mathbf{n}_\perp$ denotes the tension vector. it is possible to obtain the weak formulation.

System of equations nonlinearly coupled

The model primary unknowns can be written as a function of the shape functions and their nodal values

$$Q^h(\mathbf{x},t) = \mathbf{N}_Q(\mathbf{x})\mathbf{Q}(t), \quad \mathbf{u}^h(\mathbf{x},t) = \mathbf{N}_\mathbf{u}(\mathbf{x})\mathbf{U}(t), \tag{16}$$

where the superscript h denotes the finite element solution. The fully discrete and nonlinear algebraic system of equations is found when substituting the above expressions in the weak form (14) and (15). We choose the weighting functions equal to shape functions. Denoting the time-dependent values of the primary variables as $\mathbb{Z}$

$$\mathbb{Z} = \left(\mathbf{n}^T \; \mathbf{m}^T \; \boldsymbol{\rho}^T \; \mathbf{c}^T \; \mathbf{U}^T\right)^T, \tag{17}$$

the system of equations can be expressed as a balance of internal and external forces as follows:

$$\mathbb{F}(\mathbb{Z}_{n+1}) := \mathbb{F}^{int}(\mathbb{Z}_{n+1}) - \mathbb{F}^{ext}(\mathbb{Z}_{n+1}) = \mathbf{0}, \tag{18}$$

where the subscript $n+1$ denotes the time step on which the solution is being computed. Moreover, forces $\mathbb{F}^{int}$ and $\mathbb{F}^{ext}$ can be written in a vectorial form

$$\mathbb{F}^{int} = \Big[(\mathbf{F}_n^{int})^T \; (\mathbf{F}_m^{int})^T \; (\mathbf{F}_\rho^{int})^T \; (\mathbf{F}_c^{int})^T \; (\mathbf{F}_\mathbf{u}^{int})^T\Big]^T \tag{19}$$

$$\mathbb{F}^{ext} = \Big[(\mathbf{F}_n^{ext})^T \; (\mathbf{F}_m^{ext})^T \; (\mathbf{F}_\rho^{ext})^T \; (\mathbf{F}_c^{ext})^T \; (\mathbf{F}_\mathbf{u}^{ext})^T\Big]^T \tag{20}$$

where

$$\mathbf{F}_n^{int} = \int_\Omega \mathbf{N}_n^T \frac{\partial n}{\partial t}\, d\Omega + \int_\Omega \nabla \mathbf{N}_n^T \Big[D_n \nabla n - \frac{a_n}{(b_n+c)^2} n \nabla c - n\frac{\partial \mathbf{u}}{\partial t}\Big]\, d\Omega \tag{21}$$

$$\mathbf{F}_m^{int} = \int_\Omega \mathbf{N}_m^T \frac{\partial m}{\partial t} d\Omega - \int_\Omega \nabla \mathbf{N}_m^T m \frac{\partial \mathbf{u}}{\partial t} d\Omega \tag{22}$$

$$\mathbf{F}_\rho^{int} = \int_\Omega \mathbf{N}_\rho^T \frac{\partial \rho}{\partial t} d\Omega - \int_\Omega \nabla \mathbf{N}_\rho^T \rho \frac{\partial \mathbf{u}}{\partial t} d\Omega \tag{23}$$

$$\mathbf{F}_c^{int} = \int_\Omega \mathbf{N}_c^T \frac{\partial c}{\partial t} d\Omega + \int_\Omega \nabla \mathbf{N}_c^T \Big[D_c \nabla c - c\frac{\partial \mathbf{u}}{\partial t}\Big] d\Omega \tag{24}$$

$$\mathbf{F}_\mathbf{u}^{int} = \int_\Omega \mathbf{B}_\mathbf{u}^T \Big[\mathbf{D}_{elas}\frac{\rho}{\rho_0}\mathbf{B}_\mathbf{u}\mathbf{U} + \mathbf{D}_{visco}\mathbf{B}_\mathbf{u}\dot{\mathbf{U}} + p_{cell}(\theta)(n+\xi m)\mathbf{I}\Big] d\Omega \tag{25}$$

and

$$\mathbf{F}_n^{ext} = \int_\Omega \mathbf{N}_n^T \Big[\Big(r_n + \frac{r_{n,max}c}{C_{1/2}+c}\Big) n \big(1 - \frac{n}{K}\big) - \frac{k_{1,max}c}{C_k + c}\theta^+ n + k_2 m\Big]\, d\Omega$$

$$\mathbf{F}_m^{ext} = \int_\Omega \mathbf{N}_m^T \epsilon_r \Big(r_n + \frac{r_{n,max}c}{C_{1/2}+c}\Big) m \big(1 - \frac{m}{K}\big) + \int_\Omega \mathbf{N}_m^T \frac{k_{1,max}c}{C_k + c}\theta^+ n d\Omega$$
$$- \int_\Omega \mathbf{N}_m^T \Big[k_2 m\Big] d\Omega \tag{26}$$

$$\mathbf{F}_\rho^{ext} = \int_\Omega \mathbf{N}_\rho^T \Big[\Big(r_\rho + \frac{r_{\rho,max}c}{C_\rho + c}\Big)\frac{n+\eta_b m}{R_\rho^2+\rho^2} - d_\rho\big(n+\eta_d m\big)\rho\Big] d\Omega \tag{27}$$

$$\mathbf{F}_c^{ext} = \int_\Omega \mathbf{N}_c^T \Big[\frac{k_c(n+\zeta m)c}{\Gamma + c} - d_c c\Big] d\Omega \tag{28}$$

$$\mathbf{F}_\mathbf{u}^{ext} = -\int_\Omega \mathbf{N}_\mathbf{u}^T s\rho\mathbf{u} d\Omega \tag{29}$$

The solution of the nonlinear system of equations is obtained using a standard Newton-Raphson method

References

1. Javierre E, Moreo P, Doblare M, Garcia-Aznar JM (2009) Numerical modeling of a mechano-chemical theory for wound contraction analysis. International Journal of Solids and Structures 46: 3597-3606.

2. Olsen L, Sherratt JA, Maini PK (1995) A mechanochemical model for adult dermal wound contraction and the permanence of the contracted tissue displacement profile. Journal of theoretical biology 177: 113-128.

3. Grinnell F (1994) Fibroblasts, myofibroblasts, and wound contraction. Journal of Cell Biology 124: 401-404.

4. Chiquet M, Reneda A, Huber F, Fluck M (2003) How do fibroblasts translate mechanical signals into changes in extracellular matrix production? Matrix Biology 22: 73-80.

5. Grinnell F (2000) Fibroblast-collagen-matrix contraction: growth-factor signalling and mechanical loading. Trends in cell biology 10: 362-365.

6. Grinnell F (2003) Fibroblast biology in three-dimensional collagen matrices. Trends in cell biology 13: 264-269.

7. Tomasek J, Gabbiani G, Hinz B, Chaponnier C, Brown R (2002) Myofibroblasts and mechano-regulation of connective tissue remodelling. Nature Reviews Molecular Cell Biology 3: 349-363.

8. Hinz B, Mastrangelo D, Iselin C, Chaponnier C, Gabbiani G (2001) Mechanical tension controls granulation tissue contractile activity and myofibroblast differentiation. American Journal of Pathology 159: 1009-1020.

9. Grotendorst G, Seppa H, Kleinman H, Martin G (1981) Attachment of smooth-muscle cells to collagen and their migration toward platelet-derived growth-factor. Proceedings of the National Academy of Sciences of the United States of America-Biological Sciences 78: 3669-3672.

10. Desmouliere A, Redard M, Darby I, Gabbiani G (1995) Apoptosis mediates the decrease in cellularity during the transition between granulation-tissue and scar. American Journal of Pathology 146: 56-66.

11. Serini G, Gabbiani G (1999) Mechanisms of myofibroblast activity and phenotypic modulation. Experimental cell research 250: 273-283.

12. Roberts A, Sporn M, Assoian R, Smith J, Roche N, et al. (1986) Transforming growth factor type beta: rapid induction of fibrosis and angiogenesis in vivo and stimulation of collagen formation in vitro. Proceedings of the National Academy of Sciences of the United States of America 83: 4167-4171.

13. Moreo P, Garcia-Aznar JM, Doblare M (2008) Modeling mechanosensing and its effect on the migration and proliferation of adherent cells rid f-8256-2010. Acta Biomaterialia 4: 613-621.

14. Tranquillo R, Murray J (1992) Continuum model of fibroblast-driven wound contraction - inflammation-mediation. Journal of theoretical biology 158: 135.
